# Food insecurity, fruit and vegetable consumption, and use of the Supplemental Nutrition Assistance Program (SNAP) in Appalachian Ohio

**DOI:** 10.1371/journal.pone.0295171

**Published:** 2024-02-08

**Authors:** Lei Xu, Zoë Plakias, Andrew S. Hanks, Jennifer Garner

**Affiliations:** 1 Department of Agricultural, Environmental, and Development Economics, The Ohio State University, Columbus, OH, United States of America; 2 Department of Economics, Western Washington University, Bellingham, Washington, United States of America; 3 Department of Human Sciences, The Ohio State University, Columbus, OH, United States of America; 4 Department of Nutritional Sciences, University of Michigan School of Public Health, Columbus, OH, United States of America; University of Florida, UNITED STATES

## Abstract

Food insecurity and inadequate nutrition are two major challenges that contribute to poor health conditions among U.S. households. Ohioans continue to face food insecurity, and rates of food insecurity in rural Southeast Ohio are higher than the state average. The main purpose of this project is to evaluate the associations between Supplemental Nutrition Assistance Program (SNAP) participation and food security in rural Ohio, and to explore the association between SNAP participation and fruit/vegetable consumption. We control for food shopping patterns, such as shopping frequency, because previous research reports a significant relationship between shopping patterns and food security. To achieve our purpose, we use novel household-level data on food insecurity and SNAP participation in rural Southeast Ohio, collected during the COVID-19 pandemic. We find that people who experience higher levels of food insecurity than others are more likely to participate in SNAP, though this is likely a function of selection bias. To correct for the bias, we employ the nearest neighbor matching method to match treated (SNAP participant) and untreated (similar SNAP nonparticipant) groups. We find that participating in SNAP increases the probability of being food secure by around 26 percentage points after controlling for primary food shopping patterns. We do not find any significant association between SNAP participation and estimated intake of fruits and vegetables. This study provides policymakers with suggestive evidence that SNAP is associated with food security in rural Southeast Ohio during the pandemic, and what additional factors may mediate these relationships.

## Introduction

Food insecurity, defined by the U.S. Department of Agriculture (USDA) as “a lack of consistent access to enough food for an active and healthy life,” continues to afflict millions of Americans [1]. During the last twenty years, the percentage of U.S. households who are food insecure has never been below 10 percent. After being stable for a decade, the rate of food insecurity increased to 14.6 percent in 2008 then reached a peak of 10.5% in 2011 and in 2019 decreased to the same level as 2001 (10.5 percent of households) [2, 3]. Although overall federally-measured food insecurity was unchanged from 2019 to 2020, some subgroups, such as households with children and households with Black, non-Hispanic members, experienced increases in food insecurity and very low food security [4].

In Ohio, more than 1 in 9 individuals, including 1 in 6 children, suffered from food insecurity in 2020. However, food insecurity is more severe in Southeast Ohio counties. In Southeast Ohio, more than 1 in 7 individuals, including 1 in 5 children, struggled with food insecurity in 2020. Athens County, a part of Southeast Ohio, has one of the highest rates of food insecurity in Ohio [5]. In addition, food insecurity is understood to be correlated with poverty and unemployment [6]. In 2018, Athens County has the highest rate of individuals living in poverty in the state, 30.7%, compared to 4.1% in Delaware County, the county with the lowest poverty rate.

To address food insecurity, the federal government has implemented food assistance programs that provide nutritional support for eligible households. As the first-line defense against food insecurity, the Supplemental Nutrition Assistance Program (SNAP) is the largest federal program available to help low-income households mitigate food insecurity; SNAP is a federally-funded program administered by the states, and eligibility is generally limited to households with gross incomes up to 130% of the federal poverty line [7]. However, under Broad-Based Categorical Eligibility, multiple states allow up to 200% of the federal poverty line. Households that contain an elderly or disabled member with income under 200% poverty line could participate in SNAP. In 2020, the total federal cost of SNAP was about 85.6 billion dollars (1.3 percent of total federal spending), 35% higher than the spending in 2019 [8]. It is very important to understand the effectiveness of SNAP for different households given the magnitude of this investment.

This study evaluates the association between SNAP and food security in Southeast Ohio by using matching methods and binary logistic models. We also explore the association between SNAP participation and fruit and vegetable intake. Some challenges exist in the estimation. First, people who are more food insecure are more likely to participate in SNAP. To deal with the selection bias that arises from this, we employ the nearest neighbor matching method to match treatment (SNAP participant) and control (SNAP nonparticipants with similar characteristics) households. Second, food insecurity can be impacted by many factors, and participation in SNAP is only one of those factors. Few studies consider food purchasing patterns at food stores when studying the impacts of SNAP, and it could result in omitted variable bias for those studies that do not consider these shopping patterns if SNAP participation and shopping patterns are correlated.

There is a large body of literature exploring the role of food assistance programs in mitigating food insecurity. Some research studies find that SNAP can reduce participants’ probability of being food insecure and reduce their risk of negative health consequences [9–11]. Ratcliffe, McKernan, and Zhang (2011) use an instrumental variables (IV) approach to address selection bias and find that SNAP participation can reduce the likelihood of being food insecure by 30% and reduce the likelihood of being very food insecure by 20% [12].

Other evidence suggests that SNAP participants living in USDA-designated food deserts are 11 percentage points more likely to achieve food security than SNAP-eligible nonparticipants in the same area [13]. A census tract is defined by the USDA as a food desert if the area meets low-income and low-access thresholds; the low-income threshold is “an area that has either a poverty rate greater than or equal to 20% or a median family income not exceeding 80% of the median family income in urban areas, or 80% of the statewide median family income in the nonurban area”, and the low access threshold is “at least 500 persons and/or at least 33% of the population lives more than 1 mile from a supermarket or large grocery store (10 miles, in rural census tracts)” [1, 14]. The data we use in this paper are collected in and around Athens County, OH, where some census tracts are USDA-designated food deserts.

SNAP benefits may not be sufficient to make all SNAP participants food secure. Some studies find one-quarter of SNAP recipients remain food insecure and have difficulties affording the items in the Thrifty Food Plan (TFP), the basket of food items on which SNAP payments are based [15, 16]. Kabbani and Yazbeck (2004) found that food assistance programs do not significantly moderate food insecurity in households with children ages 5–18 [17]. In addition, few research studies focus on food insecurity in rural, low-income areas. We need further research to estimate the association between SNAP participation and food insecurity for all segments of the U.S. population, especially those with higher rates of food insecurity such as rural regions like Southeast Ohio.

Food insecurity has critical implications for households in the U.S. and other regions. Some research studies find that food insecurity correlates with higher health care costs; Berkowitz et al. (2019) observe food insecure adults spend $1,834 more on health care annually than food-secure adults in the U.S. [18]. Tarasuk et al. (2019) use rich data and find that the adjusted annual cost of prescription drugs covered by the Ontario Drug Benefit Program is 23% higher for households experiencing marginal food insecurity, 49% higher for households with moderate food insecurity, and 121% higher for households with high food insecurity relative to food secure households [19]. When these low-income households need to pay more for health care, their income available to spend on food, including fruits and vegetables (FV), may decrease.

The intake of FV and other health-promoting foods by SNAP participants is an ongoing focus of discourse related to program impacts [20, 21]. Some researchers find SNAP participants have a significantly higher intake of FV than nonparticipants. Saxe-Custack et al. (2021) find child-reported FV intakes are significantly higher among SNAP participants than nonparticipants [22]. Verghese et al. (2019) find that the impacts of SNAP participation on health are limited, though incentive programs have improved the intake of fruits and vegetables [23]. Chang et al. (2015) show that SNAP has significant and positive impacts on FV consumption for participants, but the impacts vary by family characteristics, dietary habits, living conditions, and household members’ willingness to be healthy [24]. We need more studies to determine the association between SNAP participation and FV consumption after controlling for household characteristics and primary food shopping patterns.

These research studies reveal areas for further study regarding the degree to which SNAP provides low-income households with adequate food assistance. First, we know endogeneity is an issue in estimation. Low-income households with low and very low food security are more likely to enroll in SNAP than others. Some previous research attempts to “untangle” this selection effect by using sophisticated econometric techniques. Such techniques include estimating a counterfactual to evaluate differences in food security status for several months prior to and after initial receipt of SNAP benefits [25]; comparing new entrants with people who participated in SNAP for the previous 6 months [26, 27]; and propensity score matching [28]. Nord et al. (2009) and Mabli et al. (2014) find that SNAP participation is associated with a one-third decrease of individuals being food insecure within their samples. The difference is that Nord et al. (2009) study the food security of children, while Mabli et al. (2014) focus on household food security before and after beginning to participate in SNAP by using monthly data [25, 27].

Second, omitted variable bias is potentially an issue in the existing literature. Few research studies consider primary food shopping patterns in the estimation process, while some research suggests that food-insecure households are more likely to purchase lower quality food [29, 30]. Thus, our study controls for primary food shopping patterns, including the frequency of shopping for groceries and FV from different types of stores (supercenters, convenience stores, supermarkets, and farmers markets), and miles traveled to the primary food shopping store. Third, much of the literature uses outdated data. In recent years, the government has made many changes to SNAP benefits and requirements; older data may not reflect contemporary realities. Our study uses a unique dataset collected in 2020 and 2021 in Athens County, Ohio where 30.6% of the population lived below the poverty threshold between the years 2014 and 2018 [31]. Our study adds to the literature in this space and gives policymakers more nuanced insights to help them choose robust tools to address food insecurity in low-income, rural areas. We hypothesize that SNAP participation is negatively associated with food insecurity by providing benefits for low-income households, and positively associated with FV intake.

Before exploring the association between SNAP and food security, it is important to understand some potential factors that may contribute to household food insecurity. Low income and unemployment are commonly cited household contributors [32, 33]. In addition, lack of food access is one of the potential contributing factors to food insecurity in rural areas. Food access is defined as “access by individuals to adequate resources for acquiring appropriate foods for a nutritious diet” [34]. Food access can be reflected in the types of stores people shop at and the frequency of visits to those stores. Ma et al. (2017) find that households with very low food security have a higher frequency of visiting convenience or dollar stores that have less healthful food options [35]. Bonanno and Li (2015) show that improved food access, as measured by the availability and density of food stores, can decrease the probability of adults being food insecure [36]. However, relatively few studies have considered food shopping patterns when evaluating the effect of SNAP participation on household food insecurity. In this paper, our novel data allow us to include travel distance to purchase food from the primary food store, the frequency of shopping for groceries and FV, the frequency of receiving free food from charitable organizations, and the frequency of shopping for food at different types of stores. Store types include supercenters, convenience stores, superstores, and farmers markets.

The results are consistent with some previous studies. We find evidence that selection bias exists, justifying the use of the nearest neighbor matching method (NNM). After applying NNM, we find that households participating in SNAP in the last three months are significantly more likely to be food secure than similar SNAP nonparticipants, though participating in SNAP in the last three months has no significant association with very low food security. However, we do not observe a significant association between SNAP participation and the intake of fruits and vegetables. We also do not find any significant association between monthly frequency of shopping for groceries or FV specifically and food security, but we observe that SNAP participants are more likely to be food secure than nonparticipants when they have the same shopping frequency for groceries. Shopping more frequently for groceries, and for FV, specifically, is associated with a statistically significant higher intake of FV. In addition, households shopping for groceries at superstores have a statistically significant higher intake of FV. Our findings provide additional evidence for policymakers related to the efficacy of SNAP.

## Methods

### I. Data

This study uses novel consumer survey data we collected in Southeast Ohio. The main goal of the larger project was to compare individual-level, household-level, and community-level outcomes associated with two different programs—a healthy retailer initiative and a healthy food pantry initiative–endeavoring to enhance community-level healthy food access. Along with this main analysis, the rich household-level data we collected offers the opportunity to answer a variety of other questions like those considered in this paper.

**Fig 1:**
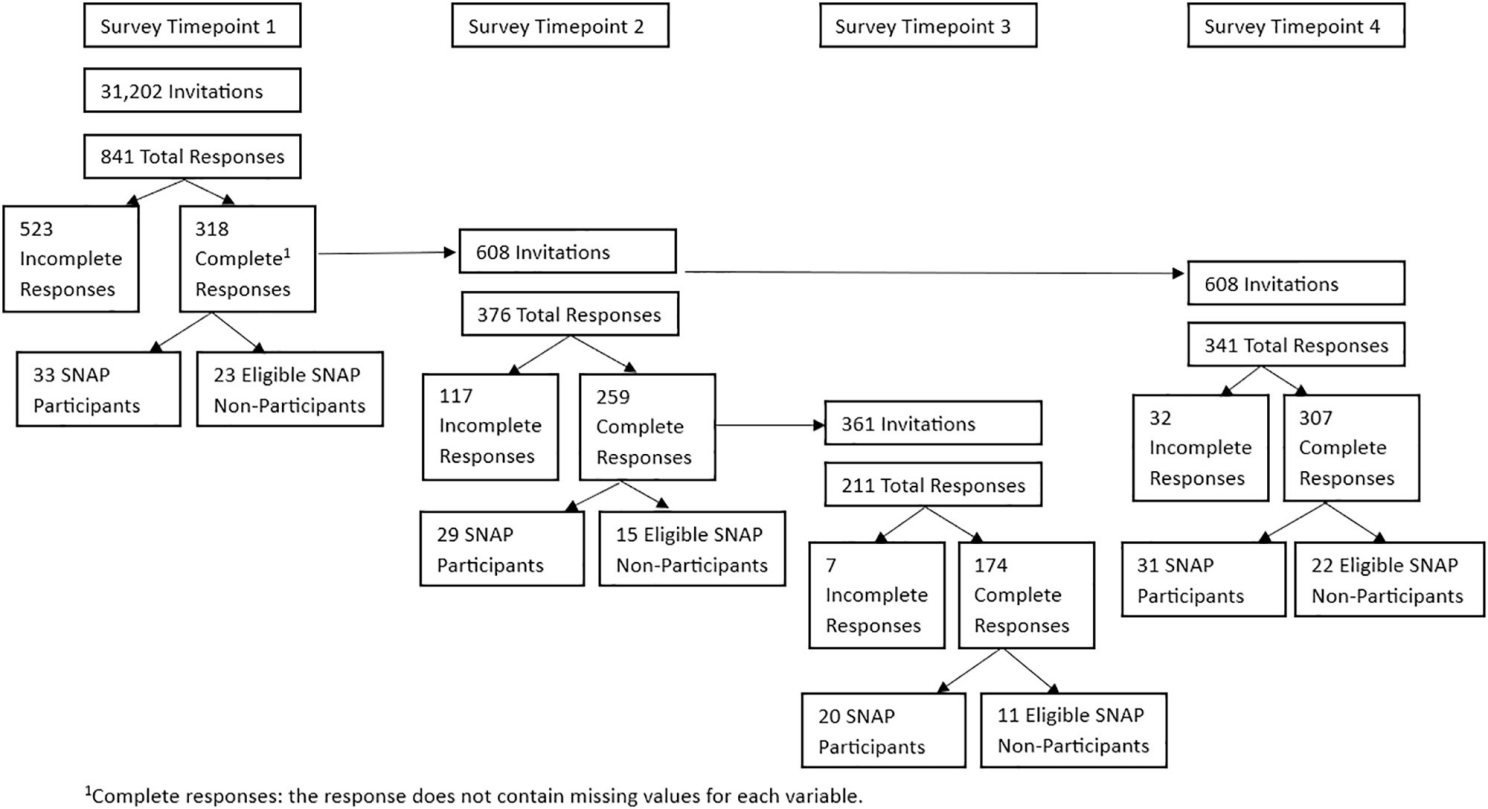
Survey timeline. ^1^Complete responses: the response does not contain missing values for each variable.

A quarterly survey was distributed via the USPS Every Door Direct Mail (EDDM) service to all 31,201 residential addresses within the 18 ZIP code regions in and around Athens County. We contracted with a third-party vendor to print and mail the postcard bundles to the applicable post offices for delivery to residential addresses in the specified zip codes. Survey respondents who wished to participate in subsequent survey rounds had to provide their contact information. All study procedures were approved by an Institutional Review Board. Study participants provided unsigned informed consent; as indicated in the informed consent script, progression to the survey online or in writing served as confirmation of informed consent. Further discussion of this sampling method and our use of it can be found in Al-Muhanna et al. (2023) [37]. One adult member per household was invited to fill out the survey. Respondents were entered into a raffle for one of 100 $25 gift cards. In addition, for those who requested a mailed pen-and-paper survey, we sent it with a pre-addressed and postage paid envelope for its return. The data were collected from 841 households (for a 2.7% response rate) via surveys. The quarterly surveys were distributed at four time points (July 2020, October 2020, January 2021, and April 2021). Although we obtained multiple time points for some households, due to attrition in data collection, we pool the observations for analysis. The survey timeline is shown in Fig 1.

To explore the representativeness of our sample, we compare our sample to relevant data from the American Community Survey (ACS). The percentage of white people in our sample is 91%, the same as the percentage of white people in Athens County in the ACS. However, compared with the ACS data, our sample has a higher median age (30.5 in ACS, 56 in our sample) and lower median income ($64,958 in ACS, $55,972 in our sample); a significant portion of people in our sample responded that they have already retired. In our sample, the proportion of people who have a bachelor’s degree or above is much higher than the proportion reported in the ACS (34.1% in ACS, 63.6% in our sample). The data differences may be because our data are more up-to-date than the ACS data, for example, many people retired in the early months of the pandemic.

**Fig 2:**
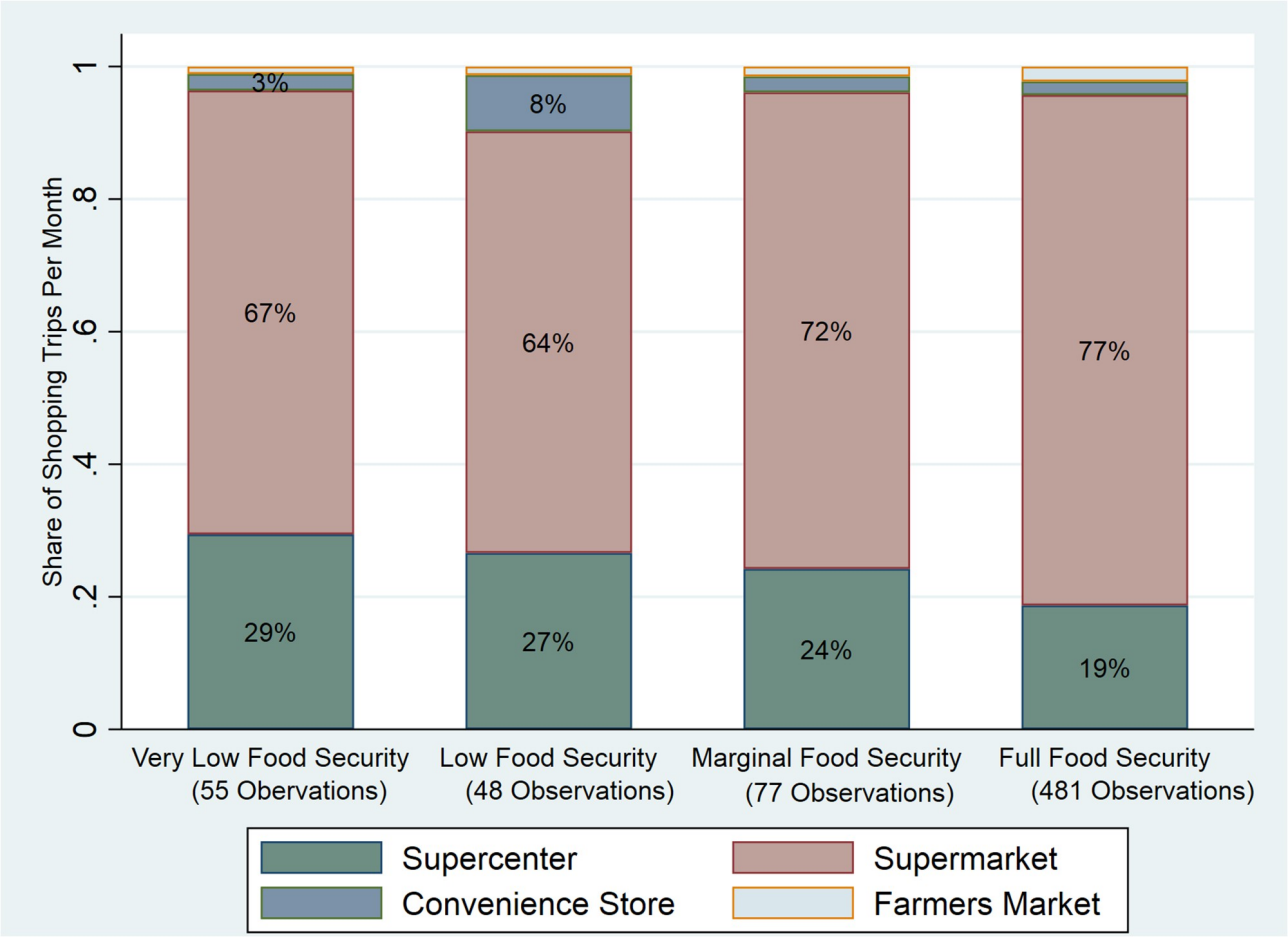
Primary food shopping location by food security status (June 2020).

The consumer survey data includes detailed household characteristics, the frequency of shopping for groceries and FV at different stores, food assistance program participation, and household food security status (See Table 1). The detailed household characteristics are adequate to determine SNAP eligibility by using household income and the number of household members. We know if a household participated in SNAP in the last three months, which gives us the opportunity to explore the association between SNAP participation and food security for households with very recent enrollment. Another strength of this consumer survey data is that primary food shopping locations are included. Based on the definitions of different stores, we put stores into different categories, including supercenters, supermarkets, convenience stores, and farmers’ markets. The definitions are based on the North American Industry Classification System definitions (see S5 Table). Different types of food stores might provide different types of foods. Farmers markets generally provide fresh produce and have some seasonal patterns in their offerings. Supermarkets provide various types of foods, and generally have more healthy food options than convenience stores. Although, the convenience stores in this region (and that were listed in the survey) were participating in Rural Action’s Country Fresh Stops program and were offering fresh produce during the growing season. Fig 2 shows the primary food shopping locations of our survey participants by different food security levels. The primary food shopping locations are the main food places where households shop for food. Households that experienced very low food security and low food security shop at convenience stores and supercenters more frequently, and shop at supermarkets less frequently than marginally food secure and fully food secure households. The differences in the frequency of visiting food stores may affect households’ food security status.

**Table 1:** Description of variables.

Variables	Description
**Independent Variables**:	
SNAP Participation 3M	1, participated in SNAP within the last 3 months; 0, no participation
SNAP Participation	1, participated in SNAP within the last 12 months; 0, no participation; this variable was only collected at survey time point 1
Age	age of the survey respondent
White	1, the respondent is white; 0, other race/ethnicity
Log of Income	log value of annual household income
Income 2020 Less	1, income earned in 2020 is less than the income earned in 2019; 0, otherwise
Num of Adults	number of adults in the household
Num of Children	number of children in the household
Any College	1, respondent has 1–3 years of college or more; 0, otherwise
Other Food Assistance 3M	1, participated in other food assistance program within the last 3 months; 0, no participation
Employed	1, employed; 0, otherwise
Unemployed	1, unemployed for 1 year or more; 0, otherwise
Travel Miles	distance traveled one way to the primary food shopping location
Freq. Grocery	the frequency of shopping for groceries at 16 commonly-visited stores per month
Freq. Charitable Grocery	the frequency of getting food from charitable sources per month
Freq. FV	the frequency of shopping for FV at 16 commonly-visited stores per month
Freq. Charitable FV	the frequency of getting FV from charitable sources per month
Survey T1	1, first survey timepoint (July 2020); 0, otherwise
Survey T2	1, second survey timepoint (October 2020); 0, otherwise
Survey T3	1, third survey timepoint (January 2021); 0, otherwise
Survey T4	1, fourth survey timepoint (April 2021); 0, otherwise
Freq. Supercenter	the frequency of shopping for groceries at supercenters per month
Freq. Convenience	the frequency of shopping for groceries at convenience stores per month
Freq. Supermarket	the frequency of shopping for groceries at supermarkets per month
Freq. Farmers	the frequency of shopping for groceries at farmers markets per month
**Dependent Variables**:	
FS(10 Item)	food security calculated by using USDA 10-item survey module; 1, food secure (full food security); 0, food insecure (marginal food security, low food security, very low food security)
FS(6 Item)	food security calculated by using USDA 6-item survey module; 1, food secure (full food security); 0, food insecure (marginal food security, low food security, very low food security)
FS(USDA)	food security calculated by using USDA 10-item survey module; 1, food secure (full food security, marginal food security); 0, food insecure (low food security, very low food security)
FV Index	fruit and vegetable index (-1.87 to 1.55)
VLFS	very low food security calculated by using USDA 10-item survey module; 1, full food security, marginal food security, low food security; 0, very low food security

Table 2 shows the summary statistics of covariates for households participating in SNAP within the last 3 months and SNAP nonparticipants (regardless of whether or not they are eligible for SNAP). On average, SNAP-participating households have lower household income, more adults and children in the household, are less likely to have attended college or higher, and are more likely to be unemployed. For primary food shopping locations, SNAP participants have a higher frequency of shopping for groceries and visiting donation sites, and they also travel a longer distance to go to primary food shopping locations than nonparticipants. The differences between SNAP participants and nonparticipants show the importance of controlling for these variables in the analysis. The last column in Table 2 shows the balance test; “N” indicates the variable is not balanced between households who participated in SNAP and households who did not participate in SNAP within the last three months and “Y” indicates the variable is balanced.

**Table 2:** Summary statistics of covariates for households participating in SNAP within the last 3 months and nonparticipants.

	Participated			Did Not Participate			Balance Test^1^
	Within the Last 3 Months			Within the Last 3 Months			
	Mean	SD	Observations	Mean	SD	Observations	
**Covariates**:							
Household Characteristics:							
Age	49.0	15.30	199	55.70	16.50	1411	N
White	0.85	0.36	199	0.92	0.28	1413	N
Income	17569	21017	186	61244	59251	1355	N
Income 2020 Less	0.08	0.27	199	0.11	0.32	1570	Y
Num of Adults	1.90	1.30	127	1.63	0.80	979	N
Num of Children	1.40	1.27	118	0.56	0.98	903	N
Any College	0.60	0.49	192	0.92	0.27	1408	N
Other Food Assistance 3M	0.51	0.50	199	0.11	0.31	1570	N
Employed	0.19	0.39	199	0.37	0.48	1570	N
Unemployed	0.07	0.25	199	0.01	0.10	1570	N
Shopping Patterns:							
Travel Miles	12.90	9.90	102	10.00	10.30	957	N
Freq. Grocery	16.10	18.10	199	10.10	9.47	1570	N
Freq. Charitable Grocery	1.08	2.62	199	0.16	1.13	1570	N
Freq. FV	11.40	14.60	199	7.40	7.25	1570	N
Freq. Charitable FV	0.86	2.31	199	0.15	1.54	1570	N
Shopping Locations:							
Freq. Supercenter	3.73	5.76	199	2.14	3.79	1570	N
Freq. Convenience	0.70	1.92	199	0.26	1.35	1570	N
Freq. Supermarket	11.60	13.30	199	7.58	7.34	1570	N
Freq. Farmers	0.22	1.78	199	0.19	1.09	1570	Y

^1^In the balance test column, N represents not balanced, Y represents balanced.

For shopping patterns, the survey question is “within the past three months, how often, on average, did you buy groceries at each location?” Survey respondents can choose one of nine options including “never,” “less than once per month,” “once per month,” “2 times per month,” “3 times per month,” “once per week,” “2–3 times per week,” “4–5 times per week,” and “6 or more times per week.” We assume 0.5 times per month on average for those who choose “less than once per month,” 10 times per month on average for those who choose “2–3 times per week,” and 18 times per month on average for those who choose “4–5 times per week.” The frequency of shopping for groceries is calculated by adding the frequency of grocery shopping at 16 commonly-visited stores in Athens County per month. The frequency of receiving food from donation sites is calculated by summing up the frequency of receiving free food from 13 commonly-visited donation sites per month. The frequency of shopping for FV is calculated by summing up the frequency of shopping for fruits and vegetables at 16 commonly-visited stores per month, and the frequency of receiving FV from donation sites is calculated by summing up the frequency of receiving fruits and vegetables from 13 commonly-visited donation sites per month.

To assess food security, the U.S. Department of Agriculture (USDA) 18-item household food security survey module was included in the consumer survey at each timepoint. Survey questions come from the USDA household food security survey module [38]. The 18-item survey has 18 questions asking about aspects of respondents’ food situations and whether they were able to afford the food they needed in the last 30 days. Among these 18 questions, 8 questions are used to measure the food situation of children. The 18-item module permits the calculation of the 10- and 6-item survey modules, since the 10- and 6-item modules are subsets of the 18-item survey module.

There are 15 occurrence questions that ask whether a specific situation associated with the experience of food insecurity ever occurred in the last 30 days and 3 number-of-occurrence questions that ask the frequency of a specific situation that occurred during the previous 1 or 2 months. Household food security status is calculated by summing up the affirmative responses, and the sum of affirmative responses composes the household’s raw score on the scale comprising those items. Affirmative responses include “yes,” “often,” “sometimes,” “almost every month,” and “some months but not every month.” Household food security status is calculated based on the survey responses. In this paper, we use food security status calculated by using 10-item and 6-item survey modules. Compared to the 18-item survey, the 10-item survey does not include 8 questions about children food situations. The 10- and 6-item survey ask about the adult food situations in the last 30 days and ask whether the household was able to afford the food needed. The 6-item survey is a subset of the 10-item survey, and it is a standard short form with known relation to the 18-item survey. We do not use the 18-item survey module because the 18-item survey module is only validated for and applicable to households with children. While all of the households in our sample have adults in them, only a portion of the households in our sample have children.

Based on the raw food security score, household food security status is generally divided into four levels: full food security, marginal food security, low food security, and very low food security [38]. Due to our relatively small sample size, in this paper we use a binary food security status that has two levels: food secure (including fully food secure households) and food insecure (including households reporting marginal food security, low food security or very low food security). For robustness, we also use a different binary food security status based on the USDA standard: food secure (including households reporting full food security or marginal food security) and food insecure (including households reporting low food security or very low food security).

In addition to food security, we are also interested in the intake of fruits and vegetables. The fruit and vegetable index, *FV*_*i*_, is created by scoring the items included in the National Cancer Institute’s Fruit and Vegetable All-Day Screener, also a subset of our consumer survey at each timepoint. The All-Day screener, a food frequency questionnaire, asks frequency and portion size questions about food items. The screener has been evaluated by some previous studies, and Thompson et al. (2002) show that the screener is useful to estimate median intake of fruit and vegetable servings in the U.S. population [39]. In the survey, we have FV intake frequency and FV intake amount for nine categories of fruits and vegetables. Based on the survey response and in accordance with standard scoring procedures [40], we first attach one value to each FV intake amount (e.g. 0.25 if households eat fruit less than 6 ounces, 0.5 if households eat fruit 6 to 10 ounces, 1 if households eat fruits 10 to 16 ounces, 1.5 if households eat fruits more than 16 ounces), then multiply the frequency with the FV intake amount. Finally, we sum up all values for nine categories of fruits and vegetables and get the intake of fruit and vegetable index after taking the log of the sum. Eq (1) shows how to calculate the FV index for each household, where *FV* is the FV index calculated based on 9 FV categories, *FVFreq*_*j*_ represents FV intake frequency for category *j*, and *FVAmount*_*j*_ represents FV intake amount for category *j*.

$$\begin{array}{c} FV=log(\sum_{j=1}^{9}FVFreq_{j}\times FVAmount_{j}) \end{array}$$ (1)

Table 3 shows the percentage of households in our sample that are food secure (full food security) and food insecure (marginal food security, low food security, and very low food security). Of the 841 households for whom we have survey data, 353 responded completely to the questions asking about their household’s food security status. For the pooled data that includes 1471 observations, 339 (23%) observations are experiencing food insecurity, and these 339 observations represent 206 households. We find 175 (11.9%) observations experience food insecurity if we categorize households based on the USDA standard (food secure—full food security, marginal food security; food insecure—low food security, and very low food security), and these 175 observations represent 112 households. The prevalence of food insecurity is similar to the national average (11.8%) in 2020 [41]. S1 Table shows the percentage of households that are food secure and food insecure at timepoint 1, and the food secure households only include households with full food security. Among all households who responded to the survey at timepoint 1, 15.6% of households reported experiencing food insecurity. The percentage is close to the food insecurity rate in Athens County from the Feeding America 2020 report, 16.9%, 5.1 percentage points higher than the U.S. average (11.8%). SNAP participants are much more likely to be food insecure than SNAP nonparticipants, with 57.3% reporting they were food insecure. This comparison shows us a strong selection effect (which occurs by design but complicates analysis)—food-insecure households are more likely to participate in SNAP.

**Table 3:** Percentage of food secure and food insecure households for different samples.

Variable	Total Households	SNAP Nonparticipants	SNAP Eligible Nonparticipants	SNAP Participants Who Participated Within the Last 3 Months	Total Households (USDA Standard)
Food Secure	77.0%	82.5%	60.6%	41.7%	88.1%
Food Insecure	23.0%	17.5%	39.4%	58.3%	11.9%
Number of Observations	1471	1265	109	187	1471

### II. Empirical approach

In our model, food insecurity is a function of SNAP participation along with household characteristics, respondent characteristics, and primary food shopping patterns. Household characteristics include the number of adults and children in each household, household income, and participation in other food assistance programs. Respondent characteristics include the age of the survey respondent, education, race and ethnicity, and employment status. Higher household earnings are likely to raise the level of household food security [42]. Some studies find a larger number of unemployed people and/or children in the household is associated with higher food insecurity [43]. Race is also correlated with income and further impacts food security [44]. In our model we use a binary variable, white, to indicate whether the survey respondent is white or not. While we recognize this binary choice provides no nuance about nonwhite people, we feel this binary variable is appropriate for our setting, which has a very high share of white people in the population overall. After choosing appropriate variables for the empirical models, we also run a correlation test. We find that miles traveled to the primary food store is strongly correlated with the variable of minutes traveled to the primary food store, with a correlation higher than 90%; we thus drop the latter variable for analysis.

To address selection bias among SNAP participants, we apply the nearest neighbor matching method (NNM) with Mahalanobis distance by using the command “MatchIt” in R. NNM is the most commonly used matching method to match treated and comparison units. The mechanism of NNM is to specify a distance between the control group and the treated group, and select the closest option in the control group to match with each option in the treated group by going through some potential options [45]. The default distance by using NNM under the command “MatchIt” is the propensity score difference [46]. However, due to some recent criticisms of propensity scores [47], we adopt Mahalanobis distance that measures distance relative to the central point, an overall mean for multivariate data [48]. Since the command “MatchIt” does not allow missing values for those variables that are matched, we lose observations with survey item nonresponse by using this method. Compared to other matching methods, NNM can produce a matched sample with a larger number of matched pairs, thus estimates can have less biased results with higher precision by using the matched sample [49]. For NNM with Mahalanobis distance, we use a set of variables to implement matching.

The variables include household income, number of adults and number of children in the household, participation in other food assistance programs, race, age, employment status, the frequency of shopping for groceries, and the frequency of shopping for groceries at convenience stores. In this paper, two different samples are used for matching. The first sample does not include the variable *travel miles*, the miles traveled to the primary food shopping location, since many survey respondents did not answer the survey question about miles traveled. To avoid losing observations, we do not include miles traveled in the first sample. The second sample includes miles traveled, resulting in a sample reduction from 148 to 86. For the first matched sample, 13 out of 18 variables are balanced after matching (See Panel A in Table 4). By using the second sample that includes miles traveled, 15 out of 19 variables are balanced (See Panel B in Table 4), but the sample size is significantly reduced. The matching process helps to pair SNAP participants with similar nonparticipants (See Panel A and Panel B in Table 4). S2 Table shows the summary statistics for all households that participated in SNAP regardless of the length of the participation.

**Table 4:** Summary statistics of covariates for households participating in SNAP within the last 3 months and nonparticipants.

	Participated			Did Not Participate			Balance Test^1^
	Within the Last 3 Months			Within the Last 3 Months			
	Mean	SD	Observations	Mean	SD	Observations	
**Panel A—Covariates: (After-match) – 1st Match**							
Household Characteristics:							
Age	39.90	11.10	74	42.20	10.20	74	Y
White	0.81	0.39	74	0.81	0.39	74	Y
Income	20832	24067	74	56595	45109	74	N
Income 2020 Less	0.08	0.28	74	0.19	0.39	74	Y
Num of Adults	1.69	1.34	74	1.55	0.92	74	Y
Num of Children	1.88	1.12	74	1.77	1.18	74	Y
Any College	0.62	0.49	74	0.91	0.30	74	N
Other Food Assistance 3M	0.74	0.44	74	0.58	0.50	74	N
Employed	0.26	0.44	74	0.41	0.49	74	Y
Unemployed	0.11	0.31	74	0.05	0.23	74	Y
Shopping Patterns:							
Freq. Grocery	18.10	22.40	74	12.30	13.40	74	Y
Freq. Charitable Grocery	1.09	3.08	74	0.22	0.87	74	N
Freq. FV	13.30	18.70	74	9.36	10.60	74	Y
Freq. Charitable FV	0.99	3.04	74	0.13	0.82	74	N
Shopping Locations:							
Freq. Supercenter	4.45	6.60	74	2.93	4.88	74	Y
Freq. Convenience	0.99	2.60	74	0.52	2.89	74	Y
Freq. Supermarket	12.30	15.90	74	8.78	11.10	74	Y
Freq. Farmers	0.39	2.79	74	0.11	0.51	74	Y
**Panel B—Covariates: (After-match) – 2nd Match**							
Household Characteristics:							
Age	40.70	10.70	43	42.00	11.60	43	Y
White	0.79	0.41	43	0.86	0.37	43	Y
Income	25883	18101	43	49837	32174	43	N
Income 2020 Less	0.16	0.37	43	0.21	0.41	43	Y
Num of Adults	1.63	0.93	43	1.70	0.83	43	Y
Num of Children	1.33	0.87	43	1.02	0.99	43	Y
Any College	0.67	0.47	43	0.86	0.35	43	N
Other Food Assistance 3M	0.77	0.43	43	0.58	0.50	43	Y
Employed	0.42	0.50	43	0.42	0.50	43	Y
Unemployed	0.05	0.21	43	0.05	0.21	43	Y
Shopping Patterns:							
Travel Miles	14.40	10.40	43	13.00	7.28	43	Y
Freq. Grocery	15.60	17.40	43	12.40	13.50	43	Y
Freq. Charitable Grocery	0.26	0.57	43	0.02	0.15	43	N
Freq. FV	10.90	16.00	43	9.30	11.30	43	Y
Freq. Charitable FV	0.24	0.62	43	0.00	0.00	43	N
Shopping Locations:							
Freq. Supercenter	4.07	6.00	43	3.36	5.04	43	Y
Freq. Convenience	0.44	0.83	43	0.21	0.69	43	Y
Freq. Supermarket	11.10	13.00	43	8.56	11.00	43	Y
Freq. Farmers	0.01	0.08	43	0.24	1.53	43	Y

^1^In balance test column, N represents not balanced, Y represents balanced.

We empirically estimate the relationship between SNAP participation and food security by using logistic regression. As a base model, Eq (2) measures the association between SNAP participation and food security. *FS*_*i*_ is the food security variable (0, food insecurity; 1, food security). We calculate two different *FS*_*i*_ variables using the 10-item and 6-item survey modules. Independent variables include whether the household participated in SNAP within the last 3 months (*SNAP*_*i*_); a vector of primary food shopping patterns (*S*_*i*_) that includes miles traveled to the primary food shopping location, the frequency of shopping for groceries or FVs per month, and the frequency of visiting charitable organizations to get free food or FVs; a vector *Z*_*i*_ of household characteristics and respondent characteristics including number of adults, number of children, income, employment status, age, race, education status, and other food assistance program participation; and a vector *T*_*i*_ indicating the last three survey rounds, T2, T3, and T4. We use coefficient estimator *β* for SNAP participants (within the last three months) to estimate the association between SNAP participation and food security. When food security (*FS*_*i*_) is replaced by very low food security (*VLFS*_*i*_; 1, full food security, marginal food security, low food security; 0, very low food security), the model is used to evaluate the relationship between SNAP participation and very low food security. To evaluate the relationship between SNAP participation and the intake of fruit and vegetables, we use an OLS regression (see Eq (3)) with the fruit and vegetable index *FV*_*i*_ as the dependent variable. Independent variables are the same as those in Eq (2) described below.

$$\begin{array}{c} log(P\left(FS_{i}\right)/\left(1-P\left(FS_{i}\right)\right)=\alpha_{1}+\beta_{1}SNAP_{i}+\rho_{1}S_{i}+\gamma_{1}Z_{i}+\theta_{1}T_{i}+\epsilon_{1i} \end{array}$$ (2)

$$\begin{array}{c} FV_{i}=\alpha_{2}+\beta_{2}SNAP_{i}+\rho_{2}S_{i}+\gamma_{2}Z_{i}+\theta_{2}T_{i}+\epsilon_{2i} \end{array}$$ (3)

## Results

The logistic regression results for the matched sample are shown in Table 5, and the results are reported as average marginal effects. Models in column (1), (3), and (4) use the matched sample from the first matching (without miles traveled), and models in column (2), (5), and (6) use the matched sample from the second matching (with travel miles). In column (1), the model has all household characteristics variables. Column (2) has one more variable that describes the distance to get food − miles traveled. After adding miles traveled to primary shopping location, the number of observations decreases to 86 from 148. To avoid losing these observations, in columns (3) and (4) we do not include miles traveled, instead we include the frequency of shopping for groceries each month and the frequency of receiving free food from charitable organizations each month in column (3), and we include the frequency of shopping for FVs and the frequency of receiving free FVs from charitable organizations each month in column (4).

**Table 5:** Marginal effects of participating in SNAP within the last 3 months on food security status (10 Item).

	*Logit Models*					
	Dependent variable: Binary Food Security Status (10 Item)					
	(1)	(2)	(3)	(4)	(5)	(6)
SNAP Participation 3M	0.245**	0.202+	0.268***	0.259**	0.210+	0.194+
	(0.081)	(0.108)	(0.079)	(0.079)	(0.111)	(0.107)
Age	−0.002	−0.010+	−0.003	−0.003	−0.010+	−0.009+
	(0.003)	(0.005)	(0.003)	(0.003)	(0.006)	(0.005)
White	−0.053	0.101	−0.068	−0.080	0.114	0.113
	(0.093)	(0.152)	(0.095)	(0.096)	(0.159)	(0.155)
Log of Income	0.245***	0.208*	0.242***	0.239***	0.211*	0.203*
	(0.055)	(0.091)	(0.054)	(0.054)	(0.091)	(0.089)
Income 2020 Less	0.051	−0.245+	0.042	0.050	−0.274+	−0.258+
	(0.103)	(0.140)	(0.102)	(0.102)	(0.146)	(0.146)
Number of Adults	0.005	−0.263***	0.018	0.015	−0.258**	−0.248**
	(0.034)	(0.077)	(0.039)	(0.039)	(0.085)	(0.084)
Number of Children	−0.052	−0.229**	−0.040	−0.045	−0.233**	−0.228**
	(0.035)	(0.088)	(0.036)	(0.035)	(0.090)	(0.087)
College	0.170+	0.139	0.129	0.141	0.129	0.143
	(0.097)	(0.121)	(0.097)	(0.098)	(0.132)	(0.125)
Other Food Assistance	−0.127	0.083	−0.125	−0.127	0.053	0.061
	(0.080)	(0.114)	(0.077)	(0.078)	(0.117)	(0.114)
Employed	0.024	0.165	0.024	0.030	0.165	0.167
	(0.078)	(0.122)	(0.078)	(0.079)	(0.121)	(0.123)
Unemployed	−0.125	0.026	−0.082	−0.100	0.058	0.077
	(0.163)	(0.205)	(0.157)	(0.158)	(0.211)	(0.208)
Travel Miles		0.010*			0.012*	0.013*
		(0.005)			(0.005)	(0.005)
Freq. Grocery			−0.003		−0.003	
			(0.003)		(0.003)	
Freq. Charitable Grocery			−0.051		0.086	
			(0.033)		(0.119)	
Freq. FV				−0.003		−0.004
				(0.003)		(0.003)
Freq. Chritable FV				−0.031		0.128
				(0.031)		(0.132)
Survey T2	0.110	0.046	0.090	0.099	−0.004	−0.010
	(0.088)	(0.113)	(0.087)	(0.087)	(0.119)	(0.115)
Survey T3	0.001	−0.131	−0.029	−0.026	−0.169	−0.161
	(0.096)	(0.136)	(0.094)	(0.095)	(0.140)	(0.142)
Survey T4	0.423***	0.326*	0.408**	0.399**	0.288*	0.283*
	(0.122)	(0.139)	(0.128)	(0.123)	(0.141)	(0.138)
Num.Obs.	148	86	148	148	86	86

^+^ p < 0.1,

* p < 0.05,

** p < 0.01,

*** p < 0.001

For all models in Table 5, the coefficient estimates of SNAP participation within the last three months are positive and in all the six models the coefficient estimate is statistically significant at the 10% level or less. The results imply that households who participated in SNAP within the last three months are more likely to be food secure than similar households who did not participate in SNAP in the last three months. For the model in column (1), participating in SNAP is associated with a 24.5 percentage point higher probability of being food secure. After we add the distance traveled to the primary food shopping store, the number of observations in column (2) changes to 86, but we still observe a positive and significant association between SNAP participation and food security. In column (3), we find that participating in SNAP within the last three months is associated with a 26.8 percentage point higher probability of being food secure, after controlling for the frequency of shopping for groceries and receiving free food from donation organizations per month. For the model in column (4), participating in SNAP is associated with a 25.9 percentage point higher probability of being food secure, after controlling for the frequency of shopping for FV, and the frequency of free FV received. Similar to the result in column (2), participating in SNAP within the last three months is associated with a higher probability of being food secure by 21.0 percentage points and 19.4 percentage points in columns (5) and (6), respectively. We also observe that having a higher income and attending college are positively associated with household food security status. In columns (2) (5) and (6), households with income in 2020 less than the previous year are more likely to experience food insecurity. We are surprised to find the distance to get food from the primary food shopping locations is positively associated with food security. Based on data limitations we cannot capture the purchasing amount of each shopping trip; purchasing amount may better reflect typical household food purchases than using shopping trip distance and frequency only. We can also observe that timepoint 4 (April 2021) has a positive and significant association with 10-item food security status and this could be due to policy shifts; it could also be due to post-lockdown economic recovery more broadly including but not limited to policy.

We also examine the interaction between SNAP participation and shopping frequency, and we find SNAP is associated with household food security among those who more frequently shop for groceries and FV (see S8 Table). This result implies that SNAP participants are more likely to be food secure than nonparticipants when they have the same frequency of shopping for groceries. As a robustness check, we also run the analysis using data from the survey at timepoint 1. The result is shown in S3 Table, and we find a positive and significant association between participating in SNAP and food security by controlling for the frequency of shopping for groceries/FV and receiving charitable groceries/FV. In addition, because we lose some observations in the matching process, we also estimate the association between SNAP participation and food security without matching using observations from all SNAP-eligible households in our data (see S6 Table). Similar to our main result, we find participation in SNAP within the last 3 months is positively associated with food security. These results are unsurprising as using this method only increases the sample by 28 (in column 1) relative to the matching process using all data from the sample. In addition to the food security status calculated by using the 10-item survey module, we also use food security status calculated by using the 6-item survey module to validate the results. S4 Table shows the logistic results for binary food security status using the 6-item survey module. The results are consistent with the results using the food security status calculated by using the 10-item survey. We also find that income and miles traveled to get food are positively associated with food security.

Table 6 shows the OLS regression results for the fruit and vegetable index. For all six models in Table 6, the coefficient estimators of SNAP participation within the last 3 months are not significant. This result implies that participating in SNAP within the last three months is not associated with higher fruit and vegetable consumption. We also find household size has a negative relationship with the intake of fruits and vegetables. Households with income less than previous year have lower FV intake than other households. Attending at least some college or above is associated with higher fruit and vegetable intake, and the marginal effects of attending college in column (1), (3), (4) and (6) are significant. We find that the frequency of shopping for groceries and the frequency of shopping for fruits and vegetables are positively associated with the intake of fruits and vegetables. In the models in columns (3) and (5), we find shopping for groceries more frequently is associated with an increase in the fruit and vegetable index by 0.5 percentage points and 0.9 percentage points respectively. In the models in columns (4) and (6), we find shopping for fruits and vegetables is associated with an increase in the fruit and vegetable index by 0.8 percentage points and 1.1 percentage points, respectively. The results are reasonable because some fruits and vegetables are fresh produce and thus more perishable, and more frequently for groceries or FVs could support household stock and consumption of perishables. To check whether the lack of a statistically significant association between SNAP participation and the FV intake is due to the loss of observations after matching, we also run the estimation by comparing SNAP participants and eligible nonparticipants. However, the estimation results are similar (see S7 Table). We also check the association between FV intake and the interactions of the frequency of shopping for groceries and FVs. We find shopping more frequently for groceries and FVs is not associated with higher FV intake, while SNAP participants who shop more frequently for groceries are associated with lower FV intake (see S10 Table).

**Table 6:** Marginal effects of participating in SNAP within the Last 3 months on fruit and vegetable index.

	OLS Regression Models					
	*Dependent variable: FV Index*					
	(1)	(2)	(3)	(4)	(5)	(6)
SNAP Participation 3M	−0.040	−0.040	−0.083	−0.085	−0.074	−0.066
	(0.078)	(0.116)	(0.077)	(0.075)	(0.114)	(0.110)
Age	0.001	−0.008	0.002	0.003	−0.006	−0.006
	(0.003)	(0.006)	(0.003)	(0.003)	(0.005)	(0.005)
White	−0.063	0.006	−0.017	0.003	−0.016	0.046
	(0.089)	(0.145)	(0.088)	(0.086)	(0.140)	(0.137)
Log of Income	−0.003	0.079	−0.017	−0.018	0.063	0.091
	(0.038)	(0.102)	(0.037)	(0.036)	(0.099)	(0.096)
Income 2020 Less	−0.181+	−0.312*	−0.183+	−0.190+	−0.263+	−0.272+
	(0.109)	(0.149)	(0.106)	(0.103)	(0.145)	(0.141)
Number of Adults	0.032	−0.189*	−0.013	−0.013	−0.182*	−0.163*
	(0.032)	(0.079)	(0.035)	(0.033)	(0.079)	(0.077)
Number of Children	−0.014	−0.126+	−0.016	−0.015	−0.126+	−0.121+
	(0.032)	(0.077)	(0.031)	(0.030)	(0.074)	(0.072)
College	0.198+	0.141	0.243*	0.255**	0.217	0.256+
	(0.102)	(0.145)	(0.100)	(0.097)	(0.151)	(0.145)
Other Food Assistance	0.062	0.263*	0.079	0.079	0.312*	0.302*
	(0.077)	(0.126)	(0.075)	(0.073)	(0.125)	(0.120)
Employed	0.094	−0.071	0.097	0.085	−0.093	−0.132
	(0.081)	(0.135)	(0.078)	(0.077)	(0.132)	(0.129)
Unemployed	0.317*	0.236	0.254+	0.244+	0.107	0.063
	(0.136)	(0.265)	(0.133)	(0.131)	(0.264)	(0.255)
Travel Miles		−0.003			−0.004	−0.005
		(0.006)			(0.006)	(0.006)
Freq. Grocery			0.005*		0.009*	
			(0.002)		(0.003)	
Freq. Charitable Grocery			0.025		−0.041	
			(0.017)		(0.130)	
Freq. FV				0.008***		0.011**
				(0.002)		(0.004)
Freq. Charitable FV				0.023		0.054
				(0.017)		(0.119)
Survey T2	0.028	0.035	0.069	0.072	0.107	0.095
	(0.088)	(0.128)	(0.086)	(0.084)	(0.129)	(0.126)
Survey T3	0.048	−0.061	0.091	0.101	0.007	0.070
	(0.101)	(0.171)	(0.099)	(0.097)	(0.168)	(0.166)
Survey T4	−0.006	−0.048	0.044	0.058	0.017	0.050
	(0.101)	(0.124)	(0.100)	(0.097)	(0.123)	(0.121)
Num.Obs.	148	86	148	148	86	86
R^2^	0.089	0.247	0.154	0.195	0.314	0.350

^+^ p < 0.1,

* p < 0.05,

** p < 0.01,

*** p < 0.001

Table 7 shows the marginal effects of the logistic regression results for very low food security. The marginal effects of SNAP participation within the last three months are not significant. This result suggests that for those participants with very low food security, the SNAP benefits may not be sufficient to lift them out of very low food security. The results are not consistent with some previous literature showing that SNAP benefits can reduce the likelihood of having very low food security by 20% [12]. For those participants with very low food security, they may need more SNAP benefits or other complementary resources to help them escape very low food security.

**Table 7:** Marginal effects of participating in SNAP within the last 3 months on very low food security status.

	Logit Models		
	*Dependent variable: Binary Very Low Food Security*		
	(1)	(2)	(3)
SNAP Participation 3M	0.021	−0.006	−0.012
	(0.068)	(0.061)	(0.060)
Age	−0.001	−0.001	−0.001
	(0.003)	(0.003)	(0.003)
White	−0.085	−0.148	−0.136
	(0.078)	(0.094)	(0.094)
Log of Income	0.154***	0.123***	0.121***
	(0.033)	(0.030)	(0.030)
Income 2020 Less	0.112	0.142	0.144
	(0.092)	(0.122)	(0.121)
Number of Adults	−0.027	−0.024	−0.028
	(0.028)	(0.026)	(0.026)
Number of Children	−0.027	−0.021	−0.021
	(0.028)	(0.028)	(0.028)
College	−0.031	−0.052	−0.052
	(0.090)	(0.075)	(0.075)
Employed	−0.018	0.004	0.006
	(0.071)	(0.073)	(0.074)
Unemployed	−0.213+	−0.154	−0.150
	(0.119)	(0.111)	(0.111)
Freq. Grocery		−0.001	
		(0.002)	
Freq. Charitable Grocery		0.009	
		(0.012)	
Freq. FV			−0.001
			(0.002)
Freq. Charitable FV			0.011
			(0.013)
Survey T2	0.077	0.071	0.073
	(0.078)	(0.068)	(0.068)
Survey T3	0.214*	0.287*	0.288*
	(0.089)	(0.131)	(0.130)
Survey T4	0.171+	0.173	0.176
	(0.089)	(0.115)	(0.115)
Num.Obs.	148	148	148

^+^ p < 0.1,

* p < 0.05,

** p < 0.01,

*** p < 0.001

To understand the association between the specific types of stores where respondents buy groceries and food security, we use four categories of food stores: supercenters, convenience stores, supermarkets, and farmers markets. Table 8 shows the logistic regression results including food sourcing patterns. In column (1) and (2), we check the association between the specific type of stores where respondents buy groceries and food security calculated by using the 10- and 6-item survey module, and we find weak evidence that participating in SNAP within the last three months increases the probability of being food secure (using the 6-item survey). The coefficients of the interaction term between SNAP participation and shopping frequency at primary food stores are not significant, except for the interaction between shop frequency at supermarkets and SNAP participation. The result in column (1) implies that SNAP participants who shop more frequently at supermarkets are more likely to be food secure by 1.5 percentage points, compared to SNAP nonparticipants who shop for groceries at supermarkets with the same shopping frequency.

**Table 8:** Marginal effects of participating in SNAP within the last 3 months by considering frequency and type of food retail shopping.

	*Dependent variable*:				
	FS (10 Item)	FS (6 Item)	FS (USDA)	VLFS	FV Index
	Logit Models				OLS Model
	(1)	(2)	(3)	(4)	(5)
SNAP Participation 3M	0.110	0.219*	0.101	0.026	−0.072
	(0.090)	(0.087)	(0.086)	(0.072)	(0.093)
Age	−0.002	−0.003	−0.003	−0.001	0.003
	(0.003)	(0.003)	(0.003)	(0.003)	(0.003)
White	−0.070	−0.065	−0.056	−0.113	0.003
	(0.099)	(0.100)	(0.096)	(0.097)	(0.092)
Log of Income	0.280***	0.206***	0.213***	0.130***	−0.024
	(0.060)	(0.052)	(0.051)	(0.034)	(0.037)
Income 2020 Less	0.036	0.055	0.023	0.119	−0.205+
	(0.101)	(0.109)	(0.103)	(0.120)	(0.110)
Num of Adults	0.000	−0.022	−0.021	−0.045	−0.023
	(0.040)	(0.039)	(0.035)	(0.029)	(0.037)
Num of Children	−0.033	−0.016	−0.023	−0.020	−0.004
	(0.036)	(0.036)	(0.035)	(0.029)	(0.033)
Other Food Assistance	−0.147+	−0.115	−0.084	−0.069	0.139+
	(0.081)	(0.081)	(0.080)	(0.069)	(0.080)
College	0.185+	0.124	0.077	−0.051	0.247*
	(0.098)	(0.095)	(0.083)	(0.076)	(0.105)
Employed	0.017	0.018	0.012	0.044	0.118
	(0.077)	(0.078)	(0.075)	(0.076)	(0.081)
Unemployed	−0.120	−0.126	−0.026	−0.138	0.276*
	(0.151)	(0.149)	(0.136)	(0.111)	(0.136)
Freq. Charitable Grocery	−0.074+	−0.098*	−0.045	−0.024	0.015
	(0.039)	(0.039)	(0.044)	(0.024)	(0.032)
Freq. Supercenter	−0.013	−0.015	−0.006	0.011	−0.002
	(0.009)	(0.009)	(0.008)	(0.020)	(0.010)
Freq. Convenience	−0.056	−0.052	−0.206	3.443	−0.020
	(0.123)	(0.143)	(0.204)	(295.837)	(0.016)
Freq. Supermarket	−0.010*	−0.004	−0.004	−0.008+	0.009*
	(0.004)	(0.004)	(0.004)	(0.005)	(0.005)
Freq. Farmers	0.176	0.366	4.472	1.467	0.070
	(0.428)	(0.551)	(350.143)	(416.516)	(0.094)
Freq. Supercenter × SNAP	0.015	0.012	0.000	−0.016	0.020
	(0.013)	(0.013)	(0.012)	(0.021)	(0.014)
Freq. Convenience × SNAP	0.042	0.030	0.191	−3.449	0.005
	(0.126)	(0.145)	(0.206)	(295.879)	(0.024)
Freq. Supermarket × SNAP	0.015*	0.008	0.008	0.010+	−0.009
	(0.006)	(0.006)	(0.006)	(0.005)	(0.006)
Freq. Farmers × SNAP	−0.159	−0.233	−4.243	−1.416	−0.050
	(0.565)	(0.552)	(350.275)	(416.517)	(0.094)
Survey T2	0.084	0.057	0.044	0.078	0.053
	(0.086)	(0.084)	(0.078)	(0.067)	(0.087)
Survey T3	−0.023	0.019	0.110	0.181+	0.090
	(0.091)	(0.092)	(0.094)	(0.098)	(0.101)
Survey T4	0.443***	0.373**	0.289*	0.160	0.030
	(0.131)	(0.138)	(0.133)	(0.106)	(0.101)
Num.Obs. R^2^	148	148	148	148	148
R^2^					0.200

^+^ p < 0.1,

* p < 0.05,

** p < 0.01,

*** p < 0.001

In column (3), we find that the association between participating in SNAP and food security when categorized according to the USDA standard is not significant. In column (4), we find that the association between SNAP participation and very low food security is not significant. We also find SNAP participants who shop frequently at supermarkets are more likely to avoid very low food security by 1 percentage point. In column (5), the marginal effect of participating in SNAP within the last three months is not significant. This result implies that participating in SNAP within the last three months is not associated with sourcing more fruits and vegetables for respondents in our matched sample. For the association between food security and the frequency of shopping at specific store types, we find shopping for groceries at supermarkets is associated with a 0.9 percentage point higher probability of being food secure. In addition, the results in columns (1) and (2) imply that one additional visit per month to get charitable food is associated with lower food security. The results suggest the variable of the frequency of visiting donation organizations may be endogenous, and the result is not entirely surprising because NNM cannot solve all endogeneity issues; indeed, after matching, this variable is still unbalanced between SNAP participants and nonparticipants.

## Conclusions

In our study of survey respondents from rural Southeast Ohio, we find participating in SNAP within the last three months is associated with higher food security within our matched sample of SNAP participants and similar SNAP nonparticipants. The results are consistent with some previous literature [9–12, 50–52]. Our results hold even when we control for shopping frequency or the frequency of receiving free foods. Through the interaction term between SNAP participation and the frequency of shopping for groceries, we observe SNAP participants are more likely to be food secure than nonparticipants when they have the same frequency of shopping for groceries. After taking into account the distance to get food from primary shopping locations, the association between SNAP participation and food security becomes smaller, around 20 percentage points. Moreover, we do not find a significant association between SNAP participation and very low food security. Although we want to be careful not to make any causal statements, this may suggest that SNAP participants are more likely to be food secure than similar nonparticipants at the margin, but that the program is not effective at lifting households out of very low food security.

We do not find a significant association between SNAP participation and the consumption of fruits and vegetables. One potential reason could be that consumer preferences are very hard to change. This finding in our study corresponds to the finding in Allcott et al. (2019) that differences in consumer food demand are major forces of nutritional inequality [53]. That said, it should be noted there are many systems level factors that can both shape and limit consumer preference and choice over the lifecourse. To encourage the consumption of fruits and vegetables, the federal government has allocated funds to programs that can provide SNAP participants additional benefits when they buy fruits and vegetables, such as the Double Up Food Bucks Program and GusNIP Nutrition Incentive Program via the Gus Schumacher Nutrition Incentive Program (GusNIP). However, the results of the association between SNAP participation and FV intake imply that if policymakers want to encourage the use of SNAP benefits for purchasing fruits and vegetables, additional efforts that can impact consumer demand may be needed to encourage SNAP participants to consume more fruits and vegetables in this region.

In addition, miles traveled are positively correlated with food security status. This finding is reasonable because individuals who are able to travel farther (i.e., have the necessary transportation) could access more stores and may engage in bulk food purchasing, both of which can support food security. We find the frequency of shopping for groceries has a positive relationship with fruit and vegetable intake. After separating the total food shopping trips into different categories of store types, we find some evidence of a positive and significant relationship between the frequency of shopping for groceries at grocery stores and the consumption of fruits and vegetables.

Finally, our work is based in a rural community, and rural communities are understudied in the SNAP literature. As a key policy tool intended to mitigate food insecurity, SNAP needs to be carefully evaluated by considering the impacts on all participants so that policymakers can choose whether to modify SNAP policies. Households can also benefit from these evaluations. Our research shows that households with very low food security struggle to escape from the trap of food insecurity despite participation in SNAP. Policymakers should pay greater attention to these households and consider more impactful strategies for elevating them out of food insecurity while also encouraging more widespread consumption of fruits and vegetables.

## Supporting information

S1 Table Table A.1: Percentage of food secure and food insecure households for different samples (USDA standard) at timepoint 1. (PDF)

S2 Table Table A.2: Summary statistics of covariates for SNAP participants and nonparticipants. (PDF)

S3 Table Table A.3: Marginal effects of participating in SNAP within the last 3 months on food security status (10 Item) at timepoint 1. (PDF)

S4 Table Table A.4: Marginal effects of participating in SNAP within the last 3 months on binary food security status (6 item). (PDF)

S5 Table Table A.5: Food source categorization. (PDF)

S6 Table Table A.6: Marginal effects of participating in SNAP within the last 3 months on food security status without matching (10 item), compared to eligible SNAP nonparticipants (household income < 130% poverty line). (PDF)

S7 Table Table A.7: Marginal effects of participating in SNAP within the last 3 months on FV intake without matching, compared to eligible SNAP nonparticipants (house-hold income < 130% poverty line). (PDF)

S8 Table Table A.8: SNAP participation and grocery/FV shop frequency. (PDF)

S9 Table Table A.9: Table A.9: Summary statistics of covariates for households participating in SNAP within the last 3 months and similar nonparticipants (household income < 130% poverty line). (PDF)

S10 Table Table A.10: FV intake and grocery/FV shop frequency. (PDF)

S1 File Southeast Ohio food security data file (CSV File). (CSV)

S2 File Codes for replication (do file). (R)
