# Supplementary material for: Food insecurity, fruit and vegetable consumption, and use of the Supplemental Nutrition Assistance Program (SNAP) in Appalachian Ohio
Source: PLoS One. 2024 Feb 8;19(2):e0295171. doi: 10.1371/journal.pone.0295171 (PMC10852251; doi:10.1371/journal.pone.0295171)
Supplement: S1 Table — (PDF) [file pone.0295171.s001.pdf]

S1 Table

Table A.1: Percentage of Food Secure and Food Insecure Households for Different Samples (USDA Standard) at Timepoint 1

Variable	SNAP Nonparticipants	Total Households	SNAP Participants	SNAP Participants Who Participated Within the Last 3 Months
Food Secure[1]	89.7%	84.4%	49.5%	50.0%
Food Insecure	10.3%	15.6%	50.5%	50.0%
Number of Observations	573	661	91	88

[1]Food security and food insecurity in this table are based on USDA standard, where Food Secure includes households with full food secure status and marginal food secure status, and Food Insecure includes households with low food secure status and very low food secure status.
