# Supplementary material for: Food insecurity, fruit and vegetable consumption, and use of the Supplemental Nutrition Assistance Program (SNAP) in Appalachian Ohio
Source: PLoS One. 2024 Feb 8;19(2):e0295171. doi: 10.1371/journal.pone.0295171 (PMC10852251; doi:10.1371/journal.pone.0295171)
Supplement: S2 Table — (PDF) [file pone.0295171.s002.pdf]

S2 Table

Table A.2: Summary Statistics of Covariates for SNAP Participants and Nonparticipants

	Participants			Non-Participants			Balance Test[1]
	Mean	SD	Observations	Mean	SD	Observations	
**Panel A - Covariates: (Pre-match)**							
Household Characteristics:							
Income	18372	24510	356	53247	59603	2244	N
Age	39	15.1	388	42.8	16.9	2344	N
White	0.83	0.37	388	0.84	0.28	2348	N
Any College	0.60	0.49	368	0.92	0.27	2340	N
Num of Adults	1.99	1.44	146	1.61	0.74	960	N
Num of Children	1.48	1.25	136	0.53	0.96	885	N
Other Food Assistance 3M	0.53	0.50	225	0.10	0.30	225	N
Employed	0.2	0.40	225	0.37	0.48	1544	N
Unemployed	0.06	0.24	225	0.01	0.09	1544	N
Shopping Patterns:							
Travel Miles	12.1	9.37	134	9.83	9.90	1126	N
Freq. Grocery	16.10	17.80	207	10.00	9.29	1386	N
Freq. Charitable Grocery	0.98	2.50	207	0.16	1.14	1386	N
Freq. FV	10.90	14.00	207	7.42	7.28	1361	N
Freq. Charitable FV	0.78	2.20	211	0.15	1.55	1365	N
Freq of Grocery Shopping of Groceries Per Month at:							
Freq. Supercenter	3.69	5.73	219	2.12	3.75	1408	N
Freq. Convenience	0.90	2.64	210	0.22	1.12	1389	N
Freq. Supermarket	11.40	13.10	211	7.53	7.24	1390	N
Freq. Farmers	0.20	1.68	212	0.20	1.10	1391	Y
**Panel B - Covariates: (After-match) - 1st Match**							
Household Characteristics:							
Income	22204	24096	88	65486	59282	88	N
Age	39.00	10.90	88	42.80	9.83	88	N
White	0.83	0.38	88	0.84	0.37	88	Y
Any College	0.66	0.48	88	0.90	0.31	88	N
Num of Adults	1.76	1.45	88	1.57	0.96	88	Y
Num of Children	1.85	1.08	88	1.72	1.09	88	Y
Other Food Assistance 3M	0.74	0.44		0.55	0.50		N
Employed	0.28	0.45	88	0.48	0.50	88	N
Unemployed	0.09	0.29	88	0.05	0.21	88	Y
Shopping Patterns:							
Freq. Grocery	17.20	21.00	88	11.40	12.30	88	N
Freq. Charitable Grocery	0.94	2.85	88	0.19	0.82	88	N
Freq. FV	12.10	17.40	88	9.05	9.74	88	Y
Freq. Charitable FV	0.84	2.81	88	0.13	0.78	88	N
Shopping Locations:							
Freq. Supercenter	4.23	6.51	88	2.56	3.91	88	N
Freq. Convenience	1.19	3.50	88	0.18	0.64	88	N
Freq. Supermarket	11.40	15.00	88	8.40	10.20	88	Y
Freq. Farmers	0.32	2.56	88	0.30	1.96	88	Y

[1] In balance test column, N represents not balanced, Y represents balanced.

Table A.2: Summary Statistics of Covariates for SNAP Participants and Non-participants (Continued)

	Participated Within the Last 3 Months			Did Not Participate Within the Last 3 Months			Balance Test[1]
	Mean	SD	Observations	Mean	SD	Observations	
**Panel C - Covariates: (After-match) – 2nd Match**							
Household Characteristics:							
log(Income)	9.97	0.63	42	10.90	0.58	42	N
Age	37.80	7.61	42	43.10	9.21	42	N
White	0.79	0.42	42	0.86	0.35	42	Y
Any College	0.76	0.43	42	0.91	0.30	42	Y
Num of Adults	1.38	0.83	42	1.52	0.74	42	Y
Num of Children	1.69	0.68	42	1.48	0.85	42	Y
Other Food Assistance 3M	0.86	0.35	42	0.43	0.50	42	N
Employed	0.41	0.50	42	0.52	0.51	42	Y
Unemployed	0.02	0.15	42	0.02	0.30	42	N
Shopping Patterns:							
Travel Miles	15.40	10.40	42	13.00	5.93	42	Y
Freq. Grocery	14.60	17.80	42	10.70	14.00	42	Y
Freq. Charitable Grocery	0.26	0.59	43	0.02	1.13	42	Y
Freq. FV	10.60	16.10	42	7.44	8.53	42	Y
Freq. Charitable FV	0.25	0.63	42	0.00	1.08	42	N
Shopping Locations							
Freq. Supercenter	4.45	6.85	42	2.01	6.01	42	Y
Freq. Convenience	0.39	0.80	42	0.11	0.65	42	Y
Freq. Supermarkets	4.45	9.75	42	3.44	8.56	42	N
Freq. Farmers	0.01	0.08	42	0.00	0.15	42	Y

[1]In balance test column, N represents not balanced, Y represents balanced.
