# Supplementary material for: Food insecurity, fruit and vegetable consumption, and use of the Supplemental Nutrition Assistance Program (SNAP) in Appalachian Ohio
Source: PLoS One. 2024 Feb 8;19(2):e0295171. doi: 10.1371/journal.pone.0295171 (PMC10852251; doi:10.1371/journal.pone.0295171)
Supplement: S3 Table — (PDF) [file pone.0295171.s003.pdf]

S3 Table

Table A.3: Marginal Effects of Participating in SNAP within The Last 3 Months on Food Security Status (10 Item) at Timepoint 1

	Logit Models		
	Dependent variable:		
	Binary Food Security Status (10 Item)		
	(1)	(2)	(3)
SNAP Participation 3M	0.100	0.221+	0.216+
	(0.129)	(0.115)	(0.115)
Age	0.000	−0.005	−0.003
	(0.005)	(0.004)	(0.004)
White	−0.066	−0.083	−0.074
	(0.143)	(0.119)	(0.120)
log of Income	0.202***	0.305***	0.326***
	(0.061)	(0.065)	(0.075)
Number of Adults	0.045	0.099*	0.080+
	(0.046)	(0.047)	(0.046)
Number of Children	−0.107*	−0.127***	−0.122**
	(0.051)	(0.038)	(0.039)
Any College	0.108	0.004	0.055
	(0.166)	(0.127)	(0.134)
Employed	0.057	−0.073	−0.081
	(0.120)	(0.101)	(0.104)
Unemployed	0.066	−0.023	−0.006
	(0.234)	(0.207)	(0.213)
Freq. Grocery		−0.002	
		(0.002)	
Freq. Charitable Grocery		−0.001	
		(0.020)	
Freq. FV			0.001
			(0.003)
Freq. Charitable FV			0.004
			(0.024)
Num.Obs.	78	78	78
AIC	109.8	88.2	88.4
BIC	135.7	116.4	116.7
Log.Lik.	−43.881	−32.083	−32.222
F	2.889		
RMSE	0.45		

+ $p < 0.1$, * $p < 0.05$, ** $p < 0.01$, *** $p < 0.001$
