# Supplementary material for: Food insecurity, fruit and vegetable consumption, and use of the Supplemental Nutrition Assistance Program (SNAP) in Appalachian Ohio
Source: PLoS One. 2024 Feb 8;19(2):e0295171. doi: 10.1371/journal.pone.0295171 (PMC10852251; doi:10.1371/journal.pone.0295171)
Supplement: S4 Table — (PDF) [file pone.0295171.s004.pdf]

S4 Table

Table A.4: Marginal Effects of Participating in SNAP within The Last 3 Months on Binary Food Security Status (6 Item)

	Logit Models					
	Dependent variable: Binary Food Security Status (6 Item)					
	(1)	(2)	(3)	(4)	(5)	(6)
SNAP Participation 3M	0.275**	0.244**	0.316***	0.304***	0.276**	0.249**
	(0.084)	(0.091)	(0.079)	(0.078)	(0.096)	(0.092)
Age	−0.003	−0.005	−0.004	−0.004	−0.006	−0.005
	(0.004)	(0.004)	(0.003)	(0.003)	(0.004)	(0.004)
White	−0.049	0.112	−0.083	−0.076	0.107	0.107
	(0.096)	(0.130)	(0.100)	(0.100)	(0.142)	(0.139)
log of Income	0.166***	0.119	0.218***	0.216***	0.140	0.126
	(0.040)	(0.084)	(0.050)	(0.050)	(0.085)	(0.084)
Number of Adults	0.017	−0.159**	0.026	0.017	−0.169*	−0.158*
	(0.035)	(0.061)	(0.036)	(0.036)	(0.066)	(0.065)
Number of Children	−0.037	−0.085	−0.054	−0.055	−0.085	−0.080
	(0.035)	(0.066)	(0.035)	(0.034)	(0.069)	(0.068)
Any College	0.186+	0.084	0.151	0.152	0.048	0.076
	(0.108)	(0.106)	(0.095)	(0.095)	(0.115)	(0.110)
Other Food Assistance 3M	−0.136+	−0.037	−0.132+	−0.128	−0.065	−0.065
	(0.080)	(0.101)	(0.078)	(0.078)	(0.105)	(0.104)
Employed	0.022	0.131	0.029	0.031	0.138	0.136
	(0.087)	(0.106)	(0.079)	(0.080)	(0.107)	(0.108)
Unemployed	−0.105	−0.088	−0.136	−0.136	−0.041	−0.041
	(0.141)	(0.153)	(0.147)	(0.148)	(0.157)	(0.156)
Travel Miles		0.013*			0.014*	0.015*
		(0.006)			(0.006)	(0.006)
Freq. Grocery			−0.002		−0.004	
			(0.003)		(0.003)	
Freq. Charitable Grocery			0.002		−0.004	
			(0.016)		(0.096)	
Freq. FV				−0.002		−0.004
				(0.003)		(0.003)
Freq. Charitable FV				0.004		0.046
				(0.017)		(0.104)
Survey T2	0.077	−0.054	0.067	0.075	−0.082	−0.087
	(0.095)	(0.104)	(0.089)	(0.089)	(0.111)	(0.109)
Survey T3	0.077	−0.090	0.041	0.050	−0.116	−0.122
	(0.109)	(0.136)	(0.098)	(0.098)	(0.138)	(0.141)
Survey T4	0.303**	0.116	0.335**	0.349**	0.087	0.095
	(0.109)	(0.121)	(0.128)	(0.127)	(0.124)	(0.123)
Num.Obs.	148	86	148	148	86	86
AIC	186.0	94.9	174.2	174.8	97.5	97.7
BIC	230.9	131.7	222.1	222.7	139.2	139.5
Log.Lik.	−77.993	−32.467	−71.085	−71.395	−31.763	−31.871
F	4.316					
RMSE	0.43					

+ p < 0.1, * p < 0.05, ** p < 0.01, *** p < 0.001
