# Supplementary material for: Food insecurity, fruit and vegetable consumption, and use of the Supplemental Nutrition Assistance Program (SNAP) in Appalachian Ohio
Source: PLoS One. 2024 Feb 8;19(2):e0295171. doi: 10.1371/journal.pone.0295171 (PMC10852251; doi:10.1371/journal.pone.0295171)
Supplement: S5 Table — (PDF) [file pone.0295171.s005.pdf]

S5 Table

Table A.5: Food Source Categorization

**Food Source Category**	Specific Food Source	NAICS code (if applicable)
**Supercenter**: retailers that sold groceries in addition to other merchandise, such as clothing, home goods, and cosmetics	Walmart	44511003 – supermarkets and other grocery (except convenience)
**Supermarket**: retailers that primarily sold groceries, but may have additionally sold a small selection of cleaning products, health and beauty products, and other home goods<br><br>This category included small grocery stores, due to a small total number of such stores and the many similarities between supermarkets and grocery stores (i.e., primarily selling groceries)	Piggly Wiggly<br>Seaman's<br>Busy Day Market<br>New Market<br>Kindred Market<br>Kroger<br>Aldi<br>Save A Lot	44511003 – supermarkets and other grocery
**Convenience Stores**: retailers with a limited stock of grocery items, particularly fresh produce, meat, and dairy<br><br>This category included gas stations, dollar stores, pharmacies, and specialty stores	Ron's<br>L&S Drive Thru<br>CeeDee Handy Mart	Not listed in NAICS database
	Coonskin Crossing	44512001 – convenience stores
	Nelsonville Emporium	45322013 – gift, novelty, and souvenir stores
	Shriver's Pharmacy	44611009 – pharmacies and drug stores
**Farmers' Market**: indoor or outdoor markets composed of a collection of small vendors, often farmers or large-scale gardeners, selling fresh produce, eggs, meat, dairy, cheese, or baked goods directly to consumers<br><br>This category included produce or farm stands and produce auctions	Chesterhill Produce Auction	42448009 – fresh fruit and vegetable merchant wholesalers
	O'Bleness	Not listed in NAICS database
**Charitable Food Source**: any no-cost food source, including food pantries, soup kitchens, and community meal sites	Salvation Army<br>Bishopville Church of Christ<br>Cats Cupboard<br>Federal Valley Resource Center<br>Torch Feed My Sheep<br>Athens Co JFS<br>Athens Catholic Community Food<br>Coolville Library<br>OU Heritage College of Osteopathy<br>Athens Co Children's Services	NA
	The Gathering Place<br>The Basset House<br>Nelsonville Community Dinner	NA
