# Supplementary material for: Food insecurity, fruit and vegetable consumption, and use of the Supplemental Nutrition Assistance Program (SNAP) in Appalachian Ohio
Source: PLoS One. 2024 Feb 8;19(2):e0295171. doi: 10.1371/journal.pone.0295171 (PMC10852251; doi:10.1371/journal.pone.0295171)
Supplement: S6 Table — (PDF) [file pone.0295171.s006.pdf]

S6 Table

Table A.6: Marginal Effects of Participating in SNAP within The Last 3 Months on Food Security Status Without Matching (10 Item), Compared to Eligible SNAP Nonparticipants (Household Income < 130% Poverty Line)

	Logit Models					
	Dependent variable:					
	Binary Food Security Status (10 Item)					
	(1)	(2)	(3)	(4)	(5)	(6)
SNAP Participation 3M	0.024	0.217+	0.062	0.035	0.188	0.195+
	(0.078)	(0.116)	(0.078)	(0.079)	(0.118)	(0.116)
Age	0.003	0.003	0.002	0.003	0.004	0.004
	(0.003)	(0.004)	(0.003)	(0.003)	(0.004)	(0.004)
White	0.103	0.170	0.101	0.095	0.156	0.169
	(0.104)	(0.153)	(0.102)	(0.104)	(0.149)	(0.150)
log of Income	0.040	0.024	0.041	0.039	0.032	0.036
	(0.030)	(0.100)	(0.029)	(0.030)	(0.101)	(0.101)
Number of Adults	−0.027	−0.085	−0.033	−0.030	−0.092	−0.092
	(0.033)	(0.061)	(0.035)	(0.034)	(0.060)	(0.060)
Number of Children	0.024	0.046	0.039	0.037	0.054	0.051
	(0.032)	(0.064)	(0.032)	(0.032)	(0.063)	(0.063)
College	0.201*	0.195	0.140	0.162+	0.182	0.185
	(0.089)	(0.145)	(0.090)	(0.090)	(0.155)	(0.148)
Other Food Assistance	−0.071	−0.164	−0.105	−0.085	−0.196	−0.192
	(0.077)	(0.125)	(0.076)	(0.077)	(0.124)	(0.123)
Employed	0.052	−0.066	0.062	0.041	−0.086	−0.087
	(0.080)	(0.120)	(0.080)	(0.080)	(0.119)	(0.118)
Unemployed	−0.456*	−0.414	−0.427*	−0.457*	−0.415	−0.452+
	(0.225)	(0.263)	(0.216)	(0.220)	(0.262)	(0.269)
Travel Miles		0.004			0.005	0.004
		(0.007)			(0.007)	(0.007)
Freq. Grocery			−0.003		0.003	
			(0.003)		(0.004)	
Freq. Charitable Grocery			−0.074+		−0.114+	
			(0.039)		(0.059)	
Freq. FV				0.000		0.004
				(0.003)		(0.005)
Freq. Charitable FV				−0.063+		−0.094+
				(0.034)		(0.050)
Survey T2	0.204*	0.144	0.174+	0.195*	0.190	0.208
	(0.089)	(0.131)	(0.090)	(0.090)	(0.133)	(0.134)
Survey T3	0.111	0.097	0.060	0.086	0.116	0.127
	(0.102)	(0.140)	(0.101)	(0.102)	(0.140)	(0.142)
Survey T4	0.273**	0.177	0.214*	0.264**	0.234+	0.241*
	(0.093)	(0.118)	(0.095)	(0.093)	(0.122)	(0.123)
Num.Obs.	176	91	173	175	89	90
AIC	236.4	132.8	228.5	235.2	130.6	131.7
BIC	280.8	170.5	278.9	285.8	172.9	174.2
Log.Lik.	−104.218	−51.412	−98.230	−101.605	−48.292	−48.868

+ p < 0.1, * p < 0.05, ** p < 0.01, *** p < 0.001
