# Supplementary material for: Food insecurity, fruit and vegetable consumption, and use of the Supplemental Nutrition Assistance Program (SNAP) in Appalachian Ohio
Source: PLoS One. 2024 Feb 8;19(2):e0295171. doi: 10.1371/journal.pone.0295171 (PMC10852251; doi:10.1371/journal.pone.0295171)
Supplement: S7 Table — (PDF) [file pone.0295171.s007.pdf]

S7 Table

Table A.7: Marginal Effects of Participating in SNAP within The Last 3 Months on FV Intake Without Matching, Compared to Eligible SNAP Nonparticipants (Household Income < 130% Poverty Line)

	Logit Models					
	Dependent variable:					
	Binary Food Security Status (10 Item)					
	(1)	(2)	(3)	(4)	(5)	(6)
SNAP Participation 3M	0.022	−0.072	0.000	−0.046	−0.130	−0.121
	(0.065)	(0.099)	(0.067)	(0.064)	(0.095)	(0.090)
Age	0.000	−0.002	0.000	0.000	−0.001	−0.002
	(0.002)	(0.004)	(0.002)	(0.002)	(0.004)	(0.004)
White	−0.012	−0.114	0.001	0.036	−0.110	−0.068
	(0.089)	(0.139)	(0.090)	(0.086)	(0.130)	(0.123)
log of Income	−0.051*	0.016	−0.052*	−0.048*	0.004	0.039
	(0.025)	(0.085)	(0.025)	(0.024)	(0.080)	(0.076)
Income 2020 Less	−0.095	−0.103	−0.082	−0.103	−0.112	−0.119
	(0.096)	(0.126)	(0.098)	(0.094)	(0.119)	(0.113)
Number of Adults	−0.003	−0.113+	−0.020	−0.039	−0.084	−0.095+
	(0.026)	(0.061)	(0.028)	(0.027)	(0.057)	(0.054)
Number of Children	0.014	−0.062	0.008	0.001	−0.047	−0.061
	(0.027)	(0.065)	(0.028)	(0.027)	(0.061)	(0.057)
College	0.156+	0.224	0.179*	0.187*	0.438**	0.395**
	(0.080)	(0.138)	(0.082)	(0.077)	(0.145)	(0.130)
Other Food Assistance	0.141*	0.184+	0.142*	0.157*	0.210*	0.220*
	(0.066)	(0.105)	(0.067)	(0.064)	(0.099)	(0.095)
Employed	−0.034	−0.073	−0.032	−0.036	−0.128	−0.139
	(0.069)	(0.108)	(0.070)	(0.067)	(0.102)	(0.097)
Unemployed	0.013	0.097	−0.002	−0.031	−0.013	−0.080
	(0.125)	(0.245)	(0.126)	(0.121)	(0.231)	(0.220)
Travel Miles		0.003			0.003	0.000
		(0.006)			(0.005)	(0.005)
Freq. Grocery			0.003		0.013***	
			(0.002)		(0.003)	
Freq. Charitable Grocery			0.012		−0.011	
			(0.013)		(0.018)	
Freq. FV				0.009***		0.016***
				(0.002)		(0.003)
Freq. Charitable FV				0.016		0.003
				(0.015)		(0.032)
Survey T2	0.026	0.086	0.052	0.067	0.152	0.189+
	(0.076)	(0.114)	(0.079)	(0.075)	(0.109)	(0.103)
Survey T3	0.024	0.010	0.058	0.084	0.096	0.147
	(0.090)	(0.127)	(0.092)	(0.087)	(0.121)	(0.116)
Survey T4	−0.088	−0.057	−0.071	−0.038	0.038	0.060
	(0.083)	(0.106)	(0.088)	(0.082)	(0.103)	(0.098)
Num.Obs.	179	90	175	176	88	88
R2	0.098	0.260	0.120	0.205	0.389	0.458

+ $p < 0.1$, * $p < 0.05$, ** $p < 0.01$, *** $p < 0.001$
