# Supplementary material for: Food insecurity, fruit and vegetable consumption, and use of the Supplemental Nutrition Assistance Program (SNAP) in Appalachian Ohio
Source: PLoS One. 2024 Feb 8;19(2):e0295171. doi: 10.1371/journal.pone.0295171 (PMC10852251; doi:10.1371/journal.pone.0295171)
Supplement: S8 Table — (PDF) [file pone.0295171.s008.pdf]

S8 Table

Table A.8: SNAP Participation and Grocery/FV Shop Frequency

	Logit Models			
	Dependent variable: Binary Food Security Status (10 Item)			
	(1)	(2)	(3)	(4)
SNAP Participation 3M	0.164+	0.158+	0.131	0.066
	(0.093)	(0.090)	(0.157)	(0.138)
Age	−0.002	−0.002	−0.011*	−0.010+
	(0.003)	(0.003)	(0.006)	(0.005)
White	−0.073	−0.133	0.086	−0.024
	(0.092)	(0.093)	(0.169)	(0.170)
log of Income	0.310***	0.319***	0.252**	0.239**
	(0.058)	(0.055)	(0.093)	(0.091)
Income 2020 Less	0.022	0.057	−0.271+	−0.231
	(0.095)	(0.097)	(0.142)	(0.144)
Number of Adults	−0.001	−0.001	−0.253**	−0.270**
	(0.038)	(0.036)	(0.087)	(0.089)
Number of Children	−0.028	−0.042	−0.208*	−0.235**
	(0.035)	(0.034)	(0.091)	(0.087)
College	0.171+	0.182*	0.134	0.119
	(0.091)	(0.089)	(0.131)	(0.120)
Other Food Assistance	−0.146*	−0.166*	−0.016	−0.020
	(0.074)	(0.077)	(0.122)	(0.123)
Employed	0.020	0.016	0.141	0.173
	(0.074)	(0.076)	(0.124)	(0.135)
Unemployed	−0.118	−0.149	0.127	0.151
	(0.143)	(0.143)	(0.215)	(0.204)
Travel Miles			0.010+	0.012*
			(0.006)	(0.005)
Freq. Grocery	−0.015***		−0.013*	
	(0.004)		(0.005)	
Freq. Grocery $\times Freq.FV$	0.000+	0.000+	0.000	0.000
	(0.000)	(0.000)	(0.000)	(0.000)
Freq. Grocery $\times SNAP$	0.012*		0.007	
	(0.005)		(0.009)	
Freq. Grocery from DS	−0.054+		0.072	
	(0.031)		(0.129)	
Freq. FV		−0.022***		−0.018**
		(0.005)		(0.006)
Freq. FV $\times SNAP$		0.017**		0.012
		(0.006)		(0.010)
Freq. FV from DS		−0.032		0.087
		(0.023)		(0.128)
Survey T2	0.093	0.133	−0.009	0.036
	(0.085)	(0.083)	(0.118)	(0.116)
Survey T3	−0.011	0.007	−0.137	−0.103
	(0.091)	(0.092)	(0.137)	(0.136)
Survey T4	0.417***	0.426***	0.297*	0.330*
	(0.124)	(0.117)	(0.136)	(0.141)
Num.Obs.	148	148	86	86

+ $p < 0.1$, * $p < 0.05$, ** $p < 0.01$, *** $p < 0.001$
