# Supplementary material for: Food insecurity, fruit and vegetable consumption, and use of the Supplemental Nutrition Assistance Program (SNAP) in Appalachian Ohio
Source: PLoS One. 2024 Feb 8;19(2):e0295171. doi: 10.1371/journal.pone.0295171 (PMC10852251; doi:10.1371/journal.pone.0295171)
Supplement: S9 Table — (PDF) [file pone.0295171.s009.pdf]

S9 Table

Table A.9 Summary Statistics of Covariates for Households Participating in SNAP within the Last 3 Months and Similar Non-participants ((Household Income < 130% Poverty Line)

	Participated Within the Last 3 Months			Similar Nonparticipants			Balance Test[1]
	Mean	SD	Obs	Mean	SD	Obs	
**Covariates:**							
Household Characteristics:							
Age	49.0	15.30	199	47.70	18.1	125	Y
White	0.85	0.36	199	0.84	0.37	125	Y
Income	17569	21017	186	13202	14835	125	Y
Income 2020 Less	0.08	0.27	199	0.14	0.34	125	Y
Num of Adults	1.90	1.30	127	1.93	1.12	125	Y
Num of Children	1.40	1.27	118	0.66	1.03	125	N
Any College	0.60	0.49	192	0.83	0.38	125	N
Other Food Assistance 3M	0.51	0.50	199	0.36	0.48	125	N
Employed	0.19	0.39	199	0.21	0.41	125	Y
Unemployed	0.07	0.25	199	0.04	0.20	125	Y
Shopping Patterns:							
Travel Miles	12.90	9.90	102	8.64	5.96	125	N
Freq. Grocery	16.10	18.10	199	11.50	11.90	125	N
Freq. Charitable Grocery	1.08	2.62	199	0.37	2.10	125	N
Freq. FV	11.40	14.60	199	6.99	8.00	125	N
Freq. Charitable FV	0.86	2.31	199	0.25	1.17	125	N
Shopping Locations:							
Freq. Supercenter	3.73	5.76	199	2.77	4.87	125	Y
Freq. Convenience	0.70	1.92	199	0.62	2.64	125	Y
Freq. Supermarket	11.60	13.30	199	8.01	8.50	125	N
Freq. Farmers	0.22	1.78	199	0.15	0.52	125	N

[1] In the balance test column, N represents not balanced, Y represents balanced.
