# Supplementary material for: Food insecurity, fruit and vegetable consumption, and use of the Supplemental Nutrition Assistance Program (SNAP) in Appalachian Ohio
Source: PLoS One. 2024 Feb 8;19(2):e0295171. doi: 10.1371/journal.pone.0295171 (PMC10852251; doi:10.1371/journal.pone.0295171)
Supplement: S10 Table — (PDF) [file pone.0295171.s010.pdf]

S10 Table

Table A.10: FV Intake and Grocery/FV Shop Frequency

	Logit Models					
	Dependent variable: FV Index					
	(1)	(2)	(3)	(4)	(5)	(6)
SNAP Participation 3M	−0.077	−0.032	−0.053	−0.058	0.003	0.030
	(0.093)	(0.141)	(0.093)	(0.093)	(0.147)	(0.145)
Age	0.003	−0.005	0.002	0.002	−0.005	−0.003
	(0.003)	(0.005)	(0.003)	(0.003)	(0.005)	(0.005)
White	−0.046	−0.036	−0.028	−0.008	−0.025	−0.060
	(0.085)	(0.128)	(0.085)	(0.086)	(0.130)	(0.131)
log of Income	0.003	0.132	−0.003	−0.004	0.138	0.155+
	(0.036)	(0.090)	(0.036)	(0.036)	(0.091)	(0.090)
Income 2020 Less	−0.188+	−0.216+	−0.187+	−0.197+	−0.207	−0.197
	(0.103)	(0.131)	(0.102)	(0.102)	(0.133)	(0.130)
Number of Adults	0.025	−0.160*	0.017	0.016	−0.148*	−0.145*
	(0.034)	(0.071)	(0.035)	(0.035)	(0.073)	(0.071)
Number of Children	−0.012	−0.133*	−0.025	−0.022	−0.135+	−0.133*
	(0.031)	(0.068)	(0.032)	(0.031)	(0.069)	(0.067)
College	0.254**	0.152	0.264**	0.263**	0.163	0.140
	(0.098)	(0.144)	(0.097)	(0.097)	(0.146)	(0.144)
Other Food Assistance	0.045	0.227*	0.053	0.061	0.217+	0.196+
	(0.074)	(0.115)	(0.073)	(0.073)	(0.117)	(0.116)
Employed	0.049	−0.151	0.047	0.054	−0.153	−0.139
	(0.078)	(0.126)	(0.078)	(0.078)	(0.128)	(0.125)
Unemployed	0.242+	0.056	0.231+	0.246+	0.020	0.127
	(0.130)	(0.236)	(0.129)	(0.129)	(0.250)	(0.240)
Travel Miles		−0.012*			−0.011+	−0.011+
		(0.005)			(0.006)	(0.005)
Freq. Grocery			0.007		0.000	
			(0.004)		(0.006)	
Freq. Grocery $\times Freq.FV$	0.000	0.000	0.000	0.000	0.000	0.000+
	(0.000)	(0.000)	(0.000)	(0.000)	(0.000)	(0.000)
Freq. Grocery $\times SNAP$	−0.014*	−0.023*	−0.020**	−0.013*	−0.027*	−0.030**
	(0.006)	(0.009)	(0.007)	(0.006)	(0.011)	(0.010)
Freq. Grocery from DS			0.021		0.132	
			(0.017)		(0.136)	
Freq. FV				0.009		−0.009
				(0.006)		(0.007)
Freq. FV $\times SNAP$	0.024**	0.030*	0.027**	0.018*	0.032*	0.032*
	(0.008)	(0.013)	(0.009)	(0.008)	(0.014)	(0.013)
Freq. FV from DS				0.024		0.199
				(0.017)		(0.125)
Survey T2	0.053	0.144	0.074	0.065	0.122	0.090
	(0.084)	(0.114)	(0.083)	(0.083)	(0.119)	(0.117)
Survey T3	0.090	0.228	0.104	0.100	0.246	0.268+
	(0.096)	(0.162)	(0.096)	(0.096)	(0.164)	(0.162)
Survey T4	0.034	0.095	0.059	0.053	0.097	0.078
	(0.096)	(0.113)	(0.097)	(0.096)	(0.115)	(0.113)
Num.Obs.	148	86	148	148	86	86
R2	0.206	0.453	0.232	0.234	0.461	0.480

+ $p < 0.1$, * $p < 0.05$, ** $p < 0.01$, *** $p < 0.001$
